# The effects of Pre-hospital Trauma Life Support (PHTLS) training program on the on-scene time interval

**DOI:** 10.1186/s12873-022-00591-y

**Published:** 2022-03-19

**Authors:** Mohammad Hossein Esmaeilzadeh, Morteza Rostamian, Davoud Khorasani-Zavareh, Fahimeh Barghi Shirazi, Marzieh Mogharab

**Affiliations:** 1 Social Development and Health Promotion Research Center, Gonabad University of Medical Sciences, Gonabad, Iran; 2 English Department, Faculty of Medicine, Social Development and Health Promotion Research Center, Gonabad University of Medical Sciences, Gonabad, Iran; 3 Department of Neurobiology, Division of Family Medicine and Primary Care, Care Sciences and Society (NVS), H1 Huddinge, Sweden; 4 Department of Health in Disasters and Emergencies, School of Health Management and Information Sciences, Iran University of Medical Sciences, Tehran, Iran; 5 Department of Emergency Medicine, School of Nursing and Midwifery, Nursing and Midwifery School, Birjand University of Medical Sciences, Birjand, Iran

**Keywords:** Trauma, Emergency medical services, Time management, Pre-hospital Trauma Life Support

## Abstract

**Background:**

Recent studies have shown that reducing pre-hospital time could improve the outcomes of trauma victims. Due to the importance of pre-hospital time management, this study aims to determine the effects of the Pre-hospital Trauma Life Support (PHTLS) training program on the on-scene time interval reduction.

**Methods:**

The PHTLS training program was implemented based on global standards for pre-hospital emergency technicians. The research tool was a questionnaire designed by the Ministry of Health and Medical Education in Iran. The mean on-scene time interval was calculated before, after and one month after the intervention in the control (*n* = 32) and experimental group (*n* = 32). The data were analyzed using SPSS.

**Results:**

The mean on-scene time interval in the target group (one month after intervention) has been significantly lower than that of the control group. Moreover, the mean and standard deviation from the on-scene time interval in the target group has been reduced from 17.6 ± 5.5 (before intervention) to 12 ± 3.8 min (one month after intervention) which was statistically significant.

**Conclusion:**

The implementation of the PHTLS training program can lead to the reduction of on-scene time interval. Therefore, considering the role of reducing on-scene time intervals on victims’ survival, the integration of the PHTLS training programs with pre-hospital emergency medical service systems seems inevitable.

## Background

Trauma is one of the most important causes of death and disability worldwide. Trauma is also the leading cause of death in people under 46 and even adolescence. This causes irreparable physical, mental, social and economic damage to society [1, 2]. Emergency medical services around the world are working to reduce mortality in trauma losses. In so doing, the focus is often on shortening pre-hospital time, which can lead to a reduction in trauma-related deaths [3]. Therefore, the concept of the golden hour is considered an essential principle in the care of trauma patients in the pre-hospital phase [4]. The golden time indicates that if the patient receives medical assistance in less than 60 min after the injury, the chances of survival will be the most possible [5]. Golden time includes different time intervals, including notification interval, activation interval, response interval, on-scene interval, and transport interval (See Table 1) [6].

**Table 1:** Defining different time intervals for providing emergency services in Iran

Time intervals	Definition
Activation interval	The time interval between receiving an emergency call and sending an ambulance
Response interval	The time interval between dispatching and the ambulance arrival to the scene
On-Scene interval	The time interval between the ambulance arriving at the scene and leaving the scene
Transport interval	The time interval between leaving the scene and reaching the emergency department
Total pre-hospital interval	The total response time, on-scene time, and transfer time

On-scene time interval is longer than the total time intervals in the pre-hospital emergency due to the wide range of emergency care and a large number of decision-making in the treatment phase. Previous studies have shown that traumatic mission was one of the vital factors associated with increasing on-scene time [7–9]. The data from the Trauma Registration System in the state of Pennsylvania, which was conducted to investigate the relationship between the distribution of pre-hospital emergency intervals and deaths, showed that long on-scene time was associated with an increased death in trauma patients. Therefore, reducing the on-scene time is an accessible factor that eases the consequences of trauma fatalities [10]. There are designed programs that can reduce on-scene time via providing systematic care and management for trauma victims in pre-hospital emergencies. The PHTLS training program goal is to minimize the on-scene time by rapidly evaluating and focusing on the Airway, Breathing, Circulation, Disability and Exposure (ABCDE) approach and considering the trauma kinetics [11, 12]. Considering time as a significant and effective factor in providing high-quality patient care, emergency centers can provide vital and immediate services to trauma victims and improve the results of the health care system [13].

The issue that provoked this study was the long-time intervals in pre-hospital emergencies in Iran. According to reports, about 60% of deaths occurred at the crash scene or on the way to the hospital, which is related to the golden hour [6]. This highlights the importance of using effective methods to reduce time intervals. Although, there are several descriptive quantitative and qualitative studies on time intervals, which has focused on describing the phenomenon of times [6, 14], so far, to our best knowledge, no report has been presented on the potential effect of the PHTLS program on the on-scene time interval reduction. Therefore, with a different perspective, this study aims to answer whether the PHTLS training program is effective in reducing the time of presence in the pre-hospital emergency on-scene time interval.

## Method

The present study is a controlled field trial and conducted to investigate the effect of the PHTLS program on the on-scene time in pre-hospital emergency services in 2019 (July 23 till August 30). The PHTLS program is a standard curriculum for pre-hospital care providers. The published articles by Emergency Medical Consultants, Inc. on care of trauma victims in pre-hospital emergencies, and the National Association of Emergency Medical Technicians website were used to design the structure, content, and scheduling of theoretical (lectures) and practical sessions (skill stations).

The theoretical part was conducted through classical methods (slides and educational videos). The first day included course introduction, scene review and initial assessment, airway management, respiration, ventilation and oxygenation, bleeding, shock and disabilities. The second day involved group discussion and review of first-day topics, secondary assessment, trauma in children and the elderly, burn, triage and cardiopulmonary resuscitation in trauma patients. Practical sessions were performed via clinical simulation method in trauma stations and in accordance with standard scenarios of the PHTLS program in trauma victims. This embraced overturning of car and throwing of the careless driver from the vehicle, second station of seizures and head trauma at home, quarrel and stabbing in a homeless camp in the city’s outskirts, traumatic brain injury and so forth. Furthermore, at this stage, in addition to the scene assessment and initial assessment techniques, instructions based on the ABCDE approach and scenario-based training were also given.

One trained instructor and one stimulated patient were used for each station, and in all stations, checklists were used to evaluate technicians and the instructors gave the necessary feedback. The sessions lasted between 20 and 160 min (a total of 16 h) over two days [11, 15–18]. All methods were carried out in accordance with relevant guidelines and regulations.

### Study area and population

This study was performed in the Pre-hospital Emergency Center of Gonabad, in the east of Iran. The strategic location and feasibility of the Gonabad Emergency Center makes it a particularly suitable center for providing pre-hospital services to trauma victims in the northeastern region of the country. The Gonabad Emergency Center received 55,939 calls in 2018, of which 14,190 resulted in dispatch. In total, 3464 of these dispatches were related to trauma missions. The calls that did not result in dispatch included repeated calls, non-emergency calls, failed calls, harassment, and counseling.

### Sampling method

**Fig. 1:**
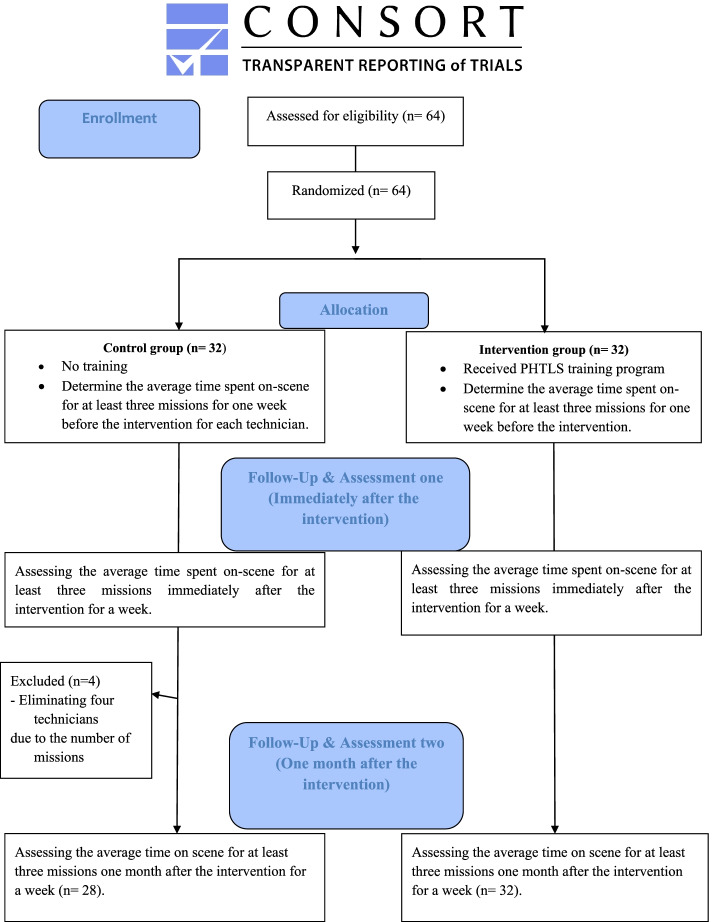
The CONSORT diagram [19]

Sampling was done in three stages through convenient, stratified and then simple random allocation. To do so, 64 technicians who met the inclusion criteria were selected via convenient sampling and divided into two categories (Associate degree and B.A). Then, samples from each level were randomly divided into experimental and control groups according to the number of technicians. It should be noted that four people in the control group were excluded from the study due to the lack of registered cases of eligible trauma victims for each technician within the specified time (See Fig. 1). Instances of trauma and on-scene time were extracted from the standard intervals of emergency services. Trauma cases were selected based on the classification of the causes of injuries based on an expanded matrix of the US Centers for Disease Control and Prevention. Consequently, trauma cases were divided into three categories: road traffic injuries, falls and other types of injuries [11].

The on-scene time interval was assessed and calculated based on the standard intervals of providing emergency services (See Table 1).

The inclusion criteria included no history of participation in the life preservation course in trauma victims, and filling the standard pre-hospital emergency care questionnaire for trauma victims who were injured and transported to the medical center by an ambulance. To achieve the average on-scene time, at least three trauma missions were extracted in three stages including time intervals before, after and one month after the intervention for each technician according to the study group (the duration of each time interval was one week and extended to two weeks if not reached the limit). To control for the confounding variables that may affect the on-scene time, only missions that required emergency medical operations were included in the study. As such missions that contained an unsafe scenes where the technicians were on hold due to scene conditions or required release (e.g. trapped patients) prior to emergency medical operations were excluded from the study.Thus, a total of 526 electronic emergency contact records that met the inclusion criteria were documented (experimental group = 272 cases; control group = 254 cases).

### Instrument

A questionnaire was used to collect the data. The first part gathered the demographic information including age, level of education and work experience of technicians, which was completed via self-report. The second part was a standard questionnaire designed by the Ministry of Health and Medical Education in Iran. It contains electronic demographic characteristics of patients, the initial diagnosis of the disease and causes of crashes, and times of emergency service delivery. Timing includes the mission announcement time, moving from the base, reaching the emergency location, leaving the emergency location, reaching the medical center, delivery to the medical center, leaving the medical center and the end of the mission time [6].

### Data analysis

Kolmogorov–Smirnov test showed that the variables are distributed normally. An independent t-test was used to compare the on-scene time interval in the experimental and control groups before and after the intervention. To compare the trend of changes in on-scene time intervals, a repeated measures ANOVA was run at the statistical level of α = 0.05. Data analysis was performed using SPSS software version 13.00 (SPSS Inc, Chicago, IL, USA).

## Results

The independent sample t-test did not show a statistically significant difference in terms of age and work experience between the two groups (*P* > 0.05; see Table 2).

**Table 2:** The distribution of demographic characteristics and causes of trauma

Demographic variables		Experimental group (*n* = 32) Percentage (Frequency)	Control group (*n* = 28) Percentage (Frequency)	*P*
Location	Urban Road	11(34.4) 21(65.6)	10(35.7) 18(64.3)	0.91
Educational level	Associate degree M.A	22(68.8) 10(31.3)	17(60.7) 11(39.3)	0.51
Trauma	Crashes Fall Other cases	187(68.8) 47(17.3) 38(14)	196(27.2) 31(12.2) 27(10.6)	0.095

Moreover, the chi-square test did not show a statistically significant difference between the experimental and control groups in personal and occupational characteristics. Therefore, the two groups were considered homogeneous.

As shown in Table 3, there was no statistically significant difference between the control and the experimental group before starting the training program.

**Table 3:** On-scene time in the experimental and control groups before, after and one month after the intervention (Time in minutes)

Group		Experimental (*n* = 32) x̄ ± SD	Control (*n* = 28) x̄ ± SD	*P*
On scene time	Time 1^a^ Time 2^b^ Time 3^c^	17.6 ± 5.5 15.1 ± 4.2 12 ± 3.8	15.6 ± 3.9 20.6 ± 20.5 19.8 ± 14.6	*P* = 0.11 *P* = 0.70 *P* = 0.05
	ANOVA	*P* < 0.001	*P* = 0.37	

Time 1^a^ = Before intervention; Time 2^b^ = After the intervention; Time 3^c^ = One month after the intervention

Similarly, there was no statistically significant difference before and after the intervention between the experimental and control groups with regard to on-scene time interval. However, on-scene time in the experimental group one month after the intervention was significantly lower than the control group (*P* = 0.05). Furthermore, on-scene time in the experimental group decreased significantly from 17.6 ± 5.5 min before the intervention to 12 ± 3.8 min one month after the intervention (*P* < 0.001).

Finally, on-scene time before, after and also one month after the intervention in the two groups were not statistically significant (*P* > 0.05) (See Table 4).

**Table 4:** On-scene time in the experimental and control group before, after and one month after the intervention (Time in minutes)

Group Time	Experimental (*n* = 32) x̄ ± SD	Control (*n* = 28) x̄ ± SD	*P*
Time 1^a^	-2.4 ± 4.8	4.8 ± 0.8	*P* = 0.057
Time 2^b^	-5.5 ± 5.2	4.2 ± 14.5	*P* < 0.001
Time 3^c^	-3 ± 5.2	-0.7 ± 24.7	*P* = 0.61

Time 1^a^ = Before and after the intervention; Time 2^b^ = Before and one month after the intervention; Time 3^c^ = After and one month after the intervention

However, there is a significant decrease in the on-scene time in the experimental group before and one month after the intervention (*P* < 0.001).

## Discussion

The present study investigated the effect of the PHTLS training program on on-scene time for pre-hospital trauma victims. The results confirmed that the PHTLS training program decreased on-scene time. The PHTLS training program with a structured approach could enhance adherence to priorities that improves trauma casualty management and the performance of technicians in dealing with a trauma casualty. Hence, the PHTLS program can possibly save time to receive definitive care. This is due to acquiring necessary skills and experience and integrating acquired training and exercises to improve decision-making skills which lead to faster transfer of trauma fatalities ([6, 11, 20]). However, it should be noted that other factors such as the skills and experience of technicians or the presence and involvement of the general public on the scene can affect the on-scene time [6].

According to Yang and Moon’s study in South Korea, the factors affecting the on-scene time are varied. This includes the instructions during resuscitation and procedures such as intubation, intravenous access, which can extend the time on-scene [21]. Therefore, reducing the on-scene time may require limiting the interventions provided in the stage, which has led to the concept of "scoop and run" for injured victims [22]. In this approach, the patient is transported quickly and safely and on-scene interventions are minimized. For example, regarding intravenous access, the emphasis is on obtaining IV in the transmission path [10].

Minimizing on-scene time with rapid assessment and focusing on the ABCDE systematic approach, trauma kinetics, and scene conditions are among the goals of the PHTLS program [11, 12]. For example, in circulatory management, establishing a venous line in the transmission path rather than at the scene of an accident can reduce on-scene time. The results of the study in the state of Michigan on the procedure of obtaining an intravenous line in a pre-hospital setting showed that starting the procedure of establishing a venous line in the transmission path is more successful than establishing a venous line at the scene of the accident [23]. This is congruent with the training provided in the PHTLS program and can be an important factor in reducing the on-scene time in the experimental group in our study.

The results of previous studies have also shown that road traffic crashes can lead to more serious injuries that may increase the on-scene time [24, 25]. According to the results of the present study, out of a total of 526 cases registered in the two groups under scrutiny, the most common cause of trauma cases is related to road traffic accidents with about 72.8%. Therefore, releasing the victims inside the car with the precautions of fixing the spine could be one of the explanations for the increased on-scene time in the present study.

Intriguingly, the on-scene time before and after implementing the PHTLS training program for trauma victims in Sweden was not significantly different between the experimental and control groups [11]. This challenges the findings of the present study in terms of the effect of educational intervention on the on-scene time. One reason could be the structural differences between the Swedish emergency system (Franco-German model) and the Iranian emergency medical service system (Anglo-American model). The provision of emergency services in these two models is different in the sense that in the Anglo-American system, the emphasis is on the rapid transfer of the patient to the medical center, but the Franco-German system is physician-centric and emphasizes the use of advanced technology and on-stage treatment. This is one of the current challenges for pre-hospital emergency systems worldwide [26]. Another eye-catching difference is that on-scene time in this study is higher than Johnson's study. This difference may be due to differences in the mechanism and type of trauma and injuries in Iran and Sweden. This could be due to the fact that most cases of trauma in Iran are traffic crashes and fatal traumas. Accordingly, in these accidents, issues such as safety of the scene, the presence of potential dangers and the release of the injured can increase the on-scene time. Therefore, before any interpretation, it is necessary to bear in mind different emergency medical care delivery models.

Another point that should be mentioned is that the result revealed that the on-scene time for the control group was increased from Time 1 to Time 2 for about five minutes. One speculation could be that there are many confounding variables in time management that are beyond the control of the researchers. For example, individual differences, psychological conditions, type of ambulance and its transportation equipment (e.g. trolleys), and scene conditions (e.g. an ambulance parked away from the accident scene). Moreover, this fact emphasizes the importance of the effects of applying training programs that not only could stop the increasing trend of on-scene time but also significantly could lead to the decrease of on-scene time. In addition to the PHTLS training program, incremental experience, repeated practice, and application effects of the training during the month might be considered as the reasons for these results.

Finally, the concept of Platinum Ten Minutes has been proposed as the necessary time to transfer the injured to the ambulance at the scene of the accident in the PHTLS training program. To achieve this goal, emergency medical personnel must coordinate with police and fire services to maintain safety at the scene and safely evacuate the injured, without causing casualties to victims or other personnel on the scene [6, 27]. However, in many emergency missions such as quarrels, fires and accidents, with the risk of explosion, emergency personnel are not allowed to enter the scene due to safety issues. Besides, due to legal gaps and challenges in criminal scenes such as robberies or suicide, disturbing the situation of the accident scene without the presence of the police or firefighters can cause problems for emergency personnel. It seems that these issues are among the factors that influence the increase of on-scene time, which deserve more future research.

## Conclusion

There are a number of decision-making factors that can influence the on-scene time, such as individual differences, and mental and psychological conditions. By random assignment of technicians in the two groups, some confounding factors were controlled. The strength of this study is that through an interventional design, we examined the impact of the PHTLS training program on pre-hospital emergency technicians.

The results of the present study showed a positive effect for the PHTLS training program and the reduction of the on-scene time for trauma victims. Since reducing the on-scene time is associated with reduced mortality and disability in trauma losses, it seems that the PHTLS training program can be used effectively for this purpose. Accordingly, we propose the integration of the PHTLS training program as an in-service and retraining program for emergency technicians.

## Data Availability

The datasets used during the current study is available from the corresponding author on reasonable request.

## Abbreviations

PHTLS: Pre-hospital Trauma Life Support
ABCDE: Airway, Breathing, Circulation, Disability and Exposure
